# Case of Asperger's Syndrome and Lesion of the Right Amygdala: Deficits in Implicit and Explicit Fearful Face Recognition

**DOI:** 10.3389/fpsyg.2021.677549

**Published:** 2021-06-22

**Authors:** Katja Koelkebeck, Jochen Bauer, Thomas Suslow, Patricia Ohrmann

**Affiliations:** Department of Psychiatry and Psychotherapy, School of Medicine, University of Muenster, Muenster, Germany

**Keywords:** amygdala, lesion, fMRI, emotion recognition, Asperger's syndrome

## Abstract

**Introduction:** Studies of brain-damaged patients revealed that amygdala lesions cause deficits in the processing and recognition of emotional faces. Patients with autism spectrum disorders (ASD) have similar deficits also related to dysfunctions of the limbic system including the amygdala.

**Methods:** We investigated a male patient who had been diagnosed with Asperger's syndrome. He also presented with a lesion of the right mesial temporal cortex, including the amygdala. We used functional magnetic resonance imaging (fMRI) to investigate neuronal processing during a passive viewing task of implicit and explicit emotional faces. Clinical assessment included a facial emotion recognition task.

**Results:** There was no amygdala activation on both sides during the presentation of masked emotional faces compared to the no-face control condition. Presentation of unmasked happy and angry faces activated the left amygdala compared to the no-face control condition. There was no amygdala activation in response to unmasked fearful faces on both sides. In the facial emotion recognition task, the patient biased positive and neutral expressions as negative.

**Conclusions:** This case report describes a male patient with right amygdala damage and an ASD. He displayed a non-response of the amygdala to fearful faces and tended to misinterpret fearful expressions. Moreover, a non-reactivity of both amygdalae to emotional facial expressions at an implicit processing level was revealed. It is discussed whether the deficient implicit processing of facial emotional information and abnormalities in fear processing could contribute and aggravate the patient's impairments in social behavior and interaction.

## Introduction

The competence to recognize emotional facial expressions and to interpret social interaction, including empathy abilities, is critical for social communication. Neuroimaging studies have provided evidence of different brain regions being responsible for facial emotion recognition (Whalen et al., 1998), such as the amygdala and the medial frontal cortices. In neurotypical individuals, the amygdala seems to be specifically activated in response to threat-related signals (fearful and angry emotional faces) (Morris et al., 2012; Todorov, 2012) especially when stimuli are masked and thus presented subliminally (Whalen et al., 1998; Suslow et al., 2006).

Patients with brain damage have repeatedly displayed deficits in emotional face recognition (Shaw et al., 2004) and empathy (Shamay-Tsoory et al., 2004) abilities. Subliminal processing of emotional stimuli is mainly performed by the right hemisphere, particularly the right amygdala, whereas conscious evaluation of emotional stimuli involves the left amygdala (Gainotti, 2012). Previous studies revealed deficient emotion processing capacities in patients with unilateral (Adolphs et al., 2002) and bilateral (Adolphs et al., 1994, 1999; Adolphs and Tranel, 2004) damage of the amygdala. This holds especially true for the processing of threat-related stimuli, whereas the recognition of happy faces seems to be undisrupted.

The observation of reduced capacities to process information of emotional or social value does not limit itself to brain damage condition. Impaired facial expression discrimination abilities have already been identified in early development of autistic children (Robel et al., 2004) and seem to persist in adult autistic individuals (Baron-Cohen et al., 2001a; Nieminen-von Wendt et al., 2005). Evidence of reduced amygdala activation during emotion processing tasks has been found in patients with Asperger's syndrome (Ashwin et al., 2007) and it is hypothesized that autistic symptoms may be caused by amygdala dysfunction (Adolphs et al., 2002). The finding of intact threat detection during simple emotional tasks (Castelli, 2005), but not during complex social situations, may indicate incorrect or dysfunctional amygdala function (Adolphs et al., 2002). Other authors attribute abnormal processing of facial emotional expressions to an unresponsiveness to the intensity of emotional expressions in patients with autism spectrum disorder (ASD) (Ashwin et al., 2007). On the other hand, autistic individuals seem to react stronger to faces expressing low intensity fear when attention is drawn toward the eye region (Lassalle et al., 2017). Adolphs and Tranel (2004) found an intact capability to process happy expressions, but as in patients with brain damage, threat-related stimuli are abnormally processed (Ogai et al., 2003; Gaigg and Bowler, 2007). Critchley et al. (2000a) reported differential deficits in brain activation in a functional neuroimaging study with ASD individuals using sub- and supraliminal emotional stimuli. Subjects with ASD showed response deficits during implicit processing of emotional facial expressions (i.e., happy and angry). In a more recent meta-analysis, Aoki et al. (2015) concluded that abnormalities in the subcortical structures, such as amygdala, hypothalamus, and basal ganglia, were associated with atypical emotional face processing in individuals with ASD.

In the case study at hand, we implemented functional magnetic resonance imaging (fMRI) to investigate neural response of the amygdala during explicit and implicit processing of different emotions, using a facial recognition tasks on a patient with early damage of the right amygdala and Asperger's syndrome. Our aim was to investigate the combined effect of cerebral damage and ASD on emotional face recognition and processing. The amygdala may form an important part of the pathogenic substrate of ASD, but it remains to be further clarified whether ASD individuals are characterized by heightened or reduced amygdala reactivity to emotional, and in particular fearful, facial expressions.

## Methods

### Case Report

**Figure 1:**
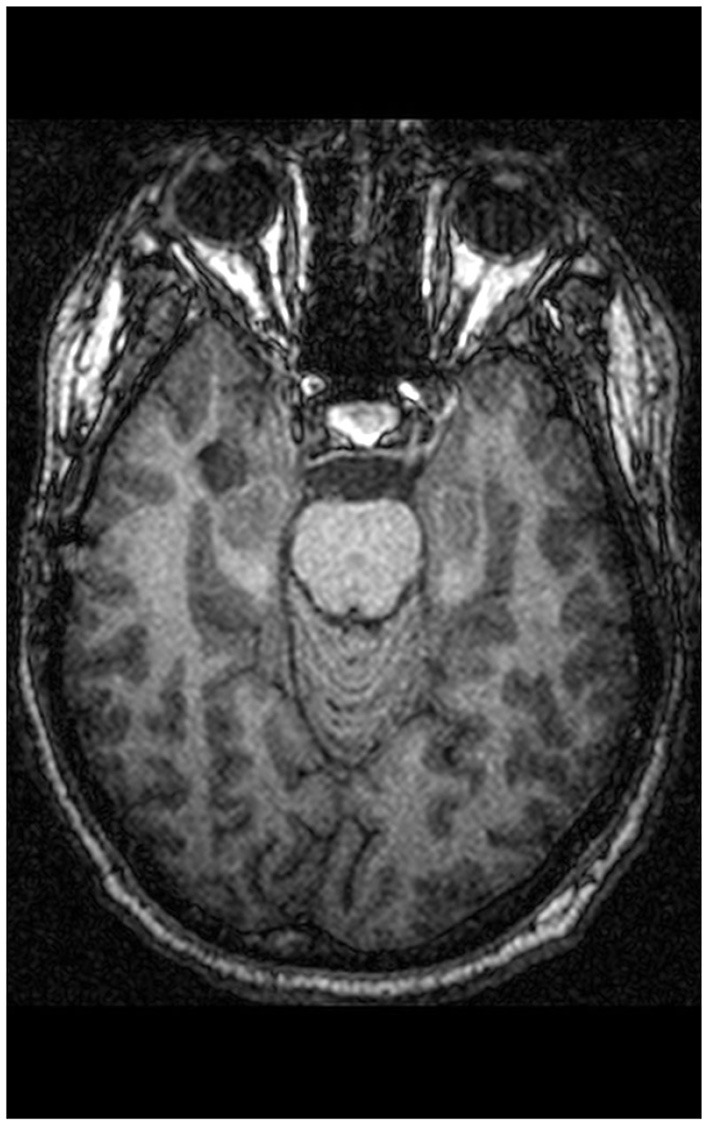
Lesion dimensions, location: temporo-basal right.

St. is a 34-year-old technician who had initially been admitted to a neurological unit because of deficits in concentration and alertness as well as recurrent alterations of consciousness. Neurological examination was normal and EEG revealed no abnormal brain waves. MRI showed a contrast medium accumulating temporo-mesial tumor, a lesion temporo-basal on the right side (22 × 6 × 12 mm) (see Figure 1). The radiologists diagnosed a low-risk tumor of the brain, a hamartoma, a dysembyoblastic neuroectodermal tumor (DNET) or ganglioglioma; the surrounding neurocranium was normal. Neuropsychological testing revealed neither deficits of language use or thinking, nor reduced visuo-constructional or practical abilities, while verbal memory deficits and executive functioning deficits (semantic and figural fluency, perseveration) were immanent. Because St. also suffered from a clinically relevant depressive mood, he was admitted to our psychiatric day-clinic. On admission, the patient complained about early awakening, depressed mood as well as mood swings; he felt easily distracted by extraneous stimuli. He reported having pressing thoughts and not being able to structure them. Furthermore, he had experienced passivity phenomena (his body felt like it was being moved from the outside). He found it difficult to get into contact with peers and felt lonely, even when in the company of others. In his recollection, concentration deficits started in elementary school. He was tense, psychomotorically agitated, and displayed manneristic behavior as well as vague speech, thought blocking, and derailment. Thinking, including abstract thinking, was normal.

According to his parents, his birth had been normal, but early in development, the patient showed abnormal behavior such as an excessive interest in technical matters. In school, the patient revealed mathematical skills above age-average, but failed in foreign languages or practical language use. Social behavior had always been abnormal; the patient had no close friends. He passed high school with an average degree and, afterwards, worked as a technician. The patient was discharged from his last employment, because he displayed concentration deficits and aggressive behavior with increasing frequency when under pressure. He had few varying facial expressions, body postures and gestures to regulate social interaction, and had difficulties developing peer relationships. He showed a stereotyped and restricted pattern of interest that was abnormal in its intensity (electric agents). He was lacking social and emotional reciprocity, could not keep himself aloof, and his sense of humor was reduced. The symptoms could not be explained by depressive mood only, since most symptoms started early in development. St. met criteria for an Asperger's syndrome (APA, 1994).

St. received 2.5 mg olanzapine and 40 mg ziprasidone at the time of testing. He had no history of substance abuse or dependence and no severe somatic disorders. The patient had no chromosomal abnormalities. His visual impairment was sufficiently corrected (Snellen eye chart). St. gave written informed consent to the experiment. It was approved by the Ethics Committee of the Medical Faculty of the University of Muenster and the Medical Association of Westphalia-Lippe and was carried out in accordance with the Declaration of Helsinki. St.'s co-operation throughout the testing sessions was good, he was very motivated. His mood was stabilized at the time of testing. St. was right-handed; handedness was defined by the Edinburgh Handedness Questionnaire (Oldfield, 1971).

### Questionnaires

The Autism Spectrum Quotient (Baron-Cohen et al., 2001b), the Empathy Quotient (Baron-Cohen and Wheelwright, 2004), and the Systemizing Quotient (Baron-Cohen et al., 2003) were administered.

### Experimental Investigation—The “Emotional Faces” Paradigm

The patient, who was naive regarding the hypotheses of the experiment, was told that he would see photographs of faces and that he should memorize them.

Face stimuli consisted of fearful, angry, happy, and neutral expressions of 10 individuals which had undergone computer gray-scale normalization. They were selected from the Pictures of Facial Affect (PFA) developed by Ekman and Friesen (1976). The PFA collection consists of 110 photographs of facial expressions that have been used in hundreds of studies, over numerous decades, to assess the ability to recognize six basic emotions within facial expressions. The facial expressions of the PFA collection express happiness, surprise, anger, fear, sadness, or disgust, or have neutral expressions. It has been solidly demonstrated that each emotion shown in the PFA collection is associated with distinct facial musculatures that are recognized by a number of cultures throughout the world (Ekman et al., 1987; Ekman, 1994). In the development of the PFA images professional actors were trained to produce emotion-related facial action configurations as defined by the Facial Action Coding System (FACS, Ekman and Friesen, 1978). The recorded facial stimuli are uniform and resemble genuine emotional facial expressions. As individuals with amygdala damage have primarily deficits in the perception of fear and individuals suffering from ASD exhibit impairments in threat-related but also other (including positive) emotions, we decided to present facial anger and fear as negative emotional expressions and facial happiness as positive emotional expression in our fMRI experiment. Moreover, we presented masked and unmasked emotional faces. In this way, we intended to assess automatic, i.e., implicit, and controlled, i.e., explicit, processing of facial expressions. In the masked face conditions we wanted to assess very rapid amygdala reactivity to covert expressions whereas in the unmasked face conditions in which faces are displayed for 500 ms we expected to assess amygdala responses to clearly visible facial stimuli. Critchley et al. (2000a) showed a neuroanatomical dissociation between implicit and explicit processing of social emotional information. The authors pointed out that a dissociation between explicit and implicit social processing appears to be apparent in patients who have intact semantic knowledge of social behavior, but who exhibit pervasive social deficits such as adults with Asperger's syndrome.

In our fMRI experiment, we administered pictures with angry, fearful, happy, and neutral emotional expressions (10 pictures per expression condition). All pictures were presented repeatedly. The patient was presented with alternating 30 s epochs of masked fearful, masked angry, masked happy, and neutral faces or a no-face control stimulus (a gray rectangle). Within epochs, masked stimuli were presented twice per second in a random sequence. Each trial had a duration of 500 ms. In masked face trials, a fearful, angry, or happy expression was presented for 33 ms, followed immediately by a 467 ms neutral expression. In the unmasked face trials of our experiment, angry, fearful, happy, and neutral facial expressions were shown for 500 ms. The no-face control stimulus was shown for 450 ms, followed by a blank screen for 50 ms. There was a counterbalanced order of presentation (Latin square design). Thus, each face epoch was preceded by a no-face control epoch and was presented twice, so that the overall presentation time was 8 min (see (Suslow et al., 2006) for details).

fMRI data were obtained while the patient was lying supine in the MR scanner tunnel. Images were projected (Sharp XG-PC10XE projector) onto a screen at the rear of the scanner tunnel which could be viewed through a mirror (8 × 12 cm) mounted at the MR head coil. Image presentation was realized by means of the software package Experimental Run Time System (ERTS; Beringer, 1999). An intelligent pre-load algorithm is built into the runtime system managing the image switching process and allowing the realization of each onset within one video refresh. The head position was stabilized by a vacuum head cushion. T2* functional data were acquired at a 3 T scanner (Gyroscan Intera 3 T, Philips Medical Systems, Best, NL) using a single shot echoplanar sequence with parameters selected to minimize distortion in the region of central interest while retaining adequate S/N and T2* sensitivity according to suggestions made by Robinson et al. (2004). Volumes consisting of 25 axial slices were acquired (matrix 128^2^, resolution 1.75 × 1.75 × 3.5 mm; TR = 3 s, TE = 30 ms, FA = 90°) 160 times in a block design, 10 times per condition.

Additionally, two anatomical datasets were acquired: T1 weighted inversion recovery and a high resolution T1 weighted 3 D sequence (isotropic pixel, 0.5 mm edge length). fMRI data were motion-corrected, in a first analysis coregistered to the anatomical dataset and in a final analysis, spatially normalized to standard MNI space (Montreal Neurological Institute) and smoothed (6 mm FWHM) using Statistical Parametric Mapping (SPM12, Wellcome Department of Cognitive Neurology, London, UK). Statistical analysis was performed by modeling the different conditions (angry, fearful, happy, neutral, and no-face) as variables within the context of the general linear model (modeled by a standard hemodynamic response function) on a voxel-by-voxel basis.

Region of interest (ROI) analysis was performed using anatomically defined ROIs for both amygdalae (left and right) using the SPM Wake Forest University (WFU) Pickatlas toolbox (http://www.fmri.wfubmc.edu/cms/software, version 3.0) (Maldjian et al., 2003). Structural MRI does not allow us to totally rule out that some amygdala neurons around the lesion remained. On the other hand, the size of the lesion should have fully covered the amygdala which can be seen if compared to the amygdala on the other side. The statistical threshold was set to *p* < 0.05 (FEW). As on this strict threshold no significant results were yielded, we applied a statistical threshold of *p* < 0.001, uncorrected.

About 15 min after the scanning session, the patient was presented with an explicit emotion recognition task which consisted of 50 emotion images of the PFA collection including previously shown stimuli and distracter stimuli (10 neutral, 10 happy, 10 angry, 10 fearful, 8 sad, and 2 disgusted expressions). He was asked (1) to decide whether he had seen the expressions on the faces during the scanning period (choose between “seen” and “not seen”; discrimination task). Task (2) was an emotion recognition/identification task. Here, the patient had to identify which emotion each face displayed (whether seen or not) and was encouraged to choose between all of the above-mentioned emotions on a list (angry, happy, fearful, disgusted, sad, neutral).

## Results

### Questionnaires

On the quotients by Baron-Cohen, he scored 31 points on the EQ (low empathy), 38 points on the SQ (average), 27 points on the AQ (autistic traits over average).

### fMRI Data

**Figure 2:**
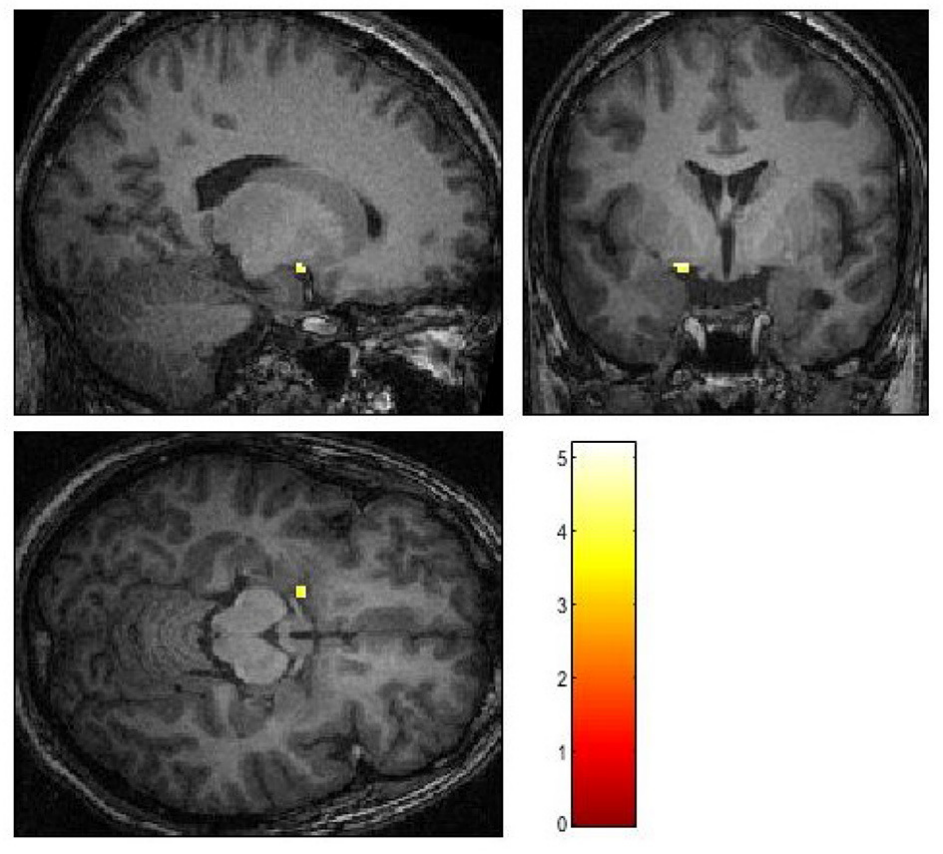
Activation patterns in reaction to angry faces in contrast to a baseline condition. *p* < 0.001, uncorrected.

Neither the coregistered nor the normalized data revealed activation in the amygdala on the side of the lesion in response to any of the stimuli. Also, no activation of either amygdala was identified in response to masked emotional stimuli or fearful stimuli in the unmasked condition. Moreover, in the unmasked condition, ROI analyses revealed a unilateral activation of the left amygdala on the contrast of angry vs. no-face condition (*X* = −18, *Y* = 0, *Z* = −12, *df* = 294, *T* = 5.19, Z-score, = 5.08, cluster size 11, *p* uncorr. < 0.001) (see Figure 2) as well as on the contrast of happy vs. no-face condition (*X* = −26, *Y* = 2, *Z* = −20, *df* = 294, *T* = 3.58, Z-score = 3.54, cluster size = 6, *p* uncorr. < 0.001).

### Discrimination Task and Emotion Recognition

In the discrimination task, St. identified 31 out of 50 stimuli correctly, i.e., he named the facial identity with the emotion it displayed correctly as “seen” or “not seen” during the scanning session. The results of the facial stimuli he falsely identified are shown in Table 1.

**Table 1:** False positive (“seen”) and false negative (“not seen”) results from the emotion discrimination task.

**Emotion**	**Neutral**	**Anger**	**Happiness**	**Fear**	**Disgust**	**Sadness**
False positive	3	4	3	4	1	–
False negative	2	2	–	–	–	–

In the emotion recognition task, St. named 30 out of 50 facial emotions correctly. He recognized all sad faces and nearly all happy faces. He had great difficulties recognizing neutral faces, identifying them falsely as “sad” in 10 out of 20 cases. In addition, in many cases angry faces were misjudged as “sad” (2 out of 8) or “disgusted” (4 out of 8), while two out of four fearful faces were also falsely identified as “disgusted.” For details, see Table 2.

**Table 2:** Results from the emotion recognition task.

**Emotion**	**Neutral**	**Happiness**	**Anger**	**Fear**	**Disgust**	**Sadness**
Neutral (20)	**10**	–	–	–	–	10
Happiness (8)	–	**7**	–	–	1	–
Anger (8)	–	–	**2**	–	4	2
Fear (4)	–	–	–	**2**	2	–
Disgust (2)	–	–	1	–	**1**	–
Sadness (8)	–	–	–	–	–	**8**

*Emotions presented are shown on the vertical axis, emotions recognized are shown on the horizontal axis. Number of correctly recognized items are printed in bold letters*.

## Discussion

In this single case study on a right amygdala lesion in an Asperger's syndrome patient, we comprehensively investigated implicit and explicit emotion recognition of faces using fMRI. The patient displayed severe deficits in emotion recognition on a behavioral level with a tendency to misinterpret neutral or angry faces.

As we here present a single-case report, results of healthy and ASD individuals without brain damage need to be compared. In a young healthy sample (age 20–40 years), stimuli taken from the Facial Expressions of Emotion Stimuli and Tests (FEEST, Young et al., 2002), which is based on images of the PFA collection and consists of 10 images per emotion condition (happiness, anger, fear, sadness, disgust, and surprise), were presented. The mean scores for recognition of emotions shown by young adults are: 9.9 (happiness), 8.2 (anger), 7.8 (fear), 8.6 (sadness), and 8.4 (disgust). This means that on average, young adults are able to correctly identify almost all happy expressions and approximately 80% of the angry and fearful expressions. Young et al. (2002) proposed cut-off scores for the FEEST defining the border between normal-range and impaired performance (for age 20–40 years; happiness: 9, anger: 5, fear: 4, sadness: 6). The recognition performance of our patient St. (happiness: 7, anger: 2, fear: 2, sadness: 8) indicates deficits in the identification of anger, fear, happiness, (but not sadness) from facial expressions compared to healthy young adults.

Moreover, we compared our patient's recognition performance with that of other patients suffering from ASD. In a meta-analysis by Lozier et al. (2014) on the impairments in facial affect recognition associated with ASD, it was found that individuals with ASD are less accurate than controls in the recognition of all six basic emotions. However, the recognition impairments were most pronounced for anger, fear, and surprise. Identification of threat-related emotions from faces could be particularly difficult in ASD. In a study based on the FEEST, the group with autism was significantly less accurate than the comparison group at recognizing fear, but not the other expressions (Humphreys et al., 2007, recognition of unambiguous expressions in experiment 1). Moreover, Enticott et al. (2014) observed that individuals with ASD were impaired in the recognition of facial expressions showing anger and disgust. Overall, it can be said that the emotion recognition impairments of St. encompass different emotional (negative and positive) expressions but are most pronounced for angry and fearful faces which is rather typical for individuals with ASD (see Lozier et al., 2014). Interestingly, in the study of Wallace et al. (2008) on facial emotion recognition, individuals with ASD confused disgust for anger. Our patient St. tended also to manifest this type of misattribution identifying falsely angry (and fearful) faces as expressions of disgust.

Data from two case reports (Calder et al., 1996; Sprengelmeyer et al., 1999) describing the effect of selective bilateral amygdala damage on facial emotion recognition show that both patients had the largest deficits in the identification of fear [2 or 5 correct responses (out of 10)], smaller deficits in the identification of anger (7 correct responses, respectively), and no impairments in the recognition of happiness (10 correct responses, respectively). In both case reports, images of the PFA collection were administered. These findings are consistent with results from a study examining the recognition of facial emotion in nine individuals with bilateral amygdala lesion (Adolphs et al., 1999). Compared to controls, the affected subjects exhibited substantial impairments in recognizing fear, were also impaired in identifying other negative emotions such as anger but no subject showed deficits in recognizing happy expressions. Against this background, it appears that the recognition deficits of our patient St. concerning facial fear could be due to his amygdala lesion. His substantial impairment in the identification of facial anger could be the result of both amygdala lesion and ASD, whereas the observed deficits in perceiving facial happiness could have emerged as part of ASD. The patient showed a bias toward the recognition of sad faces on the behavioral recognition task. Interestingly, children tend to misjudge neutral faces as sad and thus confound angry and sad faces (Golarai et al., 2006). This might reflect the ambiguity of neutral faces but might also be related to brain development disturbances (in ASD).

As regards findings from fMRI, St. did not activate the left amygdala in response to implicit emotional stimuli. While the patient showed unilateral activation to explicit emotions of anger and happiness, he did not activate the left amygdala in response to masked and unmasked fearful faces. It is interesting that this patient also tended to misidentify fearful faces. To rapidly identify fear in others is very important for self-protection as well as for prosocial behavior (Marsh et al., 2007). Individuals who more accurately identify fear are more willing to help others in distress.

Our results are in line with studies that investigated implicit emotion-processing in ASD. In a study using event-related potentials in children with high-functioning ASD (Batty et al., 2011), implicit facial emotion presentation evoked latencies and less strong amplitudes in comparison to neurotypical children. Also, neuroimaging studies in ASD individuals revealed activation deficits in response to implicit emotional stimuli (Critchley et al., 2000b). In a neuroimaging study that assessed neural activation patterns to varying degrees of emotional valence, Deeley et al. (2007) showed that patients with high-functioning ASD generally activated face perception areas less than neurotypical controls on all basic emotions (including fear). Thus, we replicated the findings of deficits in implicit emotion recognition in ASD suggesting that the amygdala is implicated in social-emotional behavior and representation of motivational meaning of faces. Critchley et al. (2000b) propose that deficits on the autonomic processing level might partly explain social impairment in ASD.

Disturbed processing of threat-related stimuli has repeatedly been related to ASD (Adolphs and Tranel, 2004). Leung et al. (2014) found in a magnetic encephalography study a lower accuracy in the recognition of angry faces and the utilization of different brain regions for happy and angry faces. Lastly, a study found that in ambiguous emotion representations with morphed pictures, a deficit in the recognition of anger, happiness and disgust was evident in ASD (Humphreys et al., 2007). However, in contrast to angry and happy facial stimuli, the patient in our case report did not respond to explicit fearful facial expressions. In an EEG study (Van der Donck et al., 2019), a reduced sensitivity of school-age children with ASD to fearful stimuli was identified, showing a delayed neural response. This is in line with findings that basic emotion identification is intact, but more complex tasks produce recognition errors in ASD (Castelli, 2005). Interestingly, one study (Vladeanu et al., 2012) reported that the discrimination of fear was lateralized to the right hemisphere in patients with ASD, which in this case might have interfered with the damage of the right amygdala. Gainotti (2012) proposed that the right amygdala is primarily responsible for automatic emotion processing. In case of right amygdala damage, a response to implicit emotional stimuli would thus not be possible, as seen in the patient in this case report.

One point of discussion might concern the attentiveness of the patient during the fMRI session. Even though our experiment required the patient to memorize the faces, but did not assess any activity (e.g., button-press) in the scanner, such an instruction should have promoted attentive behavior. This can also be deduced from his recognition performance. Another point is whether psychopharmacological treatment at the time of testing might have had an effect on the patient's performance. Our patient received antipsychotic as well as antidepressive medication at the time of the testing, which might both have affected cognitive performance. However, research shows that antipsychotic and antidepressive medication have a beneficial or non-significant effect on cognition, at least in patient groups that regularly take antipsychotics (Penn et al., 2009) or antidepressants (Dalili et al., 2015). As the patient was on a stable, low dose of both medicaments, we assume that the additional cognitive effects are to be considered low.

Our case report relates to the hypothesis that a unilateral lesion of the amygdala might be responsible for the development of autistic deficits. The damage to the right amygdala leading to a neural dysfunction regarding emotional face recognition might have impaired the incidental learning of recognizing emotion expressions in faces. However, a study of Paul et al. (2010) showed in two cases with early amygdala lesion no development of any autistic symptoms. More research in similar cases is therefore warranted.

## Conclusions

We presented a case report of a patient with unilateral amygdala damage and ASD. He displayed clinical and neuronal abnormalities in processing social information, with a tendency to misinterpret fearful expressions and a non-response of the left amygdala to fearful faces. In that, he showed features of both ASD individuals and such with amygdala damage. Moreover, the patient showed neural non-reactivity of the left amygdala to emotional facial expressions at an implicit processing level, which has also been demonstrated in ASD individuals. This case demonstrates that both ASD and amygdala damage might have affected emotion recognition abilities differently in the patient, both on the clinical and on the neuronal level. An intriguing question is if the damage to the right amygdala has worsened ASD symptoms in the patient. Impaired automatic processing of social emotional information could amplify deficits in social competence.

## Data Availability Statement

The raw data supporting the conclusions of this article will be made available by the authors, without undue reservation.

## Ethics Statement

The studies involving human participants were reviewed and approved by the Ethics Committee of the Medical Faculty of the University of Muenster and the Medical Association of Westphalia-Lippe. The patients/participants provided their written informed consent to participate in this study. Written informed consent was obtained from the individual(s) for the publication of any potentially identifiable images or data included in this article.

## Author Contributions

KK: conception, writing of draft, correspondence, data collection and analysis, and testing JB: conception, data management, data analysis, technical support, and writing the draft TS: testing, conception, providing the paradigm, writing the draft, and data analysis PO: conception, testing, and writing the draft. All authors contributed to the article and approved the submitted version.

## Conflict of Interest

The authors declare that the research was conducted in the absence of any commercial or financial relationships that could be construed as a potential conflict of interest.
